# Evaluation of the effectiveness of combined femtosecond laser-assisted cataract surgery and femtosecond laser astigmatic keratotomy in improving post-operative visual outcomes

**DOI:** 10.1186/s12886-018-0823-1

**Published:** 2018-07-03

**Authors:** Jing Wang, Jiangyue Zhao, Jun Xu, Jinsong Zhang

**Affiliations:** Department of Ophthalmology, the Fourth Affiliated Hospital of China Medical University, Eye Hospital of China Medical University,The Key Lenticular Laboratory of Liaoning Province, Shenyang, 110005 China

**Keywords:** Femtosecond laser-assisted cataract surgery, Femtosecond laser astigmatic keratotomy, Astigmatism, Effectiveness

## Abstract

**Background:**

To determine postoperative refractive and visual outcomes and astigmatic changes after femtosecond laser astigmatic keratotomy in femtosecond laser-assisted cataract surgery (FLACS).

**Methods:**

This was a prospective interventional case series. Patients with age-related cataract and corneal astigmatism (1.0–3.0D) were treated with FLACS and femtosecond laser astigmatic keratotomy (FSAK). All patients underwent examinations before and 3 months after surgery; visual acuity, subjective and objective refraction, and corneal astigmatism were evaluated and recorded for all patients by using an OPD-Scan III topographer. Vector analysis of astigmatic changes was performed by using the Alpins vector method.

**Results:**

Twenty-five patients were included in the study. Postoperatively, refractive and corneal astigmatism were both reduced significantly (*P* < 0.05), concurrent with improved uncorrected distance visual acuity and corrected distance visual acuity. The rate of spectacle use was significantly reduced at 3 months postoperatively (*P* = 0.001). The mean magnitude of the target-induced astigmatism vector (1.40 ± 0.37D) was slightly higher than the mean magnitude of the surgically induced astigmatism vector (1.22 ± 0.46D). The magnitude of error (− 0.18 ± 0.36D), as well as the correction index (0.88 ± 0.29), demonstrated slight undercorrection. The angle of error was 0.85 ± 13.69°, which was close to zero.

**Conclusions:**

Combined femtosecond laser-assisted cataract surgery and astigmatic keratotomy may be an effective approach to manage preoperative astigmatism in cataract surgery, although slight undercorrection may exist during short-term follow-up.

**Trial registration:**

ChiCTR-TRC-14004977

## Background

Modern cataract surgery has gradually evolved into a precise science, both for restoring visual acuity and for achieving emmetropia. Between 18 and 25% of patients undergoing cataract surgery exhibit > 1.5 diopters (D) of corneal astigmatism; 34–48% have > 1.0D [1, 2]. Naturally occurring and residual corneal astigmatism after cataract surgery both cause reduced uncorrected visual acuity and can contribute to glare, monocular diplopia, asthenopia, and other symptoms, leading to patient dissatisfaction. Treating preexisting corneal astigmatism to help meet patients’ demands for complete, spectacle-free visual rehabilitation is a challenge for the modern ophthalmic surgeon.

Two major techniques for the correction of preexisting corneal astigmatism during cataract surgery are corneal incision and toric intraocular lens (IOL) implantation [3–5]. Primary corneal incisions (PIs) at the steeper corneal meridian, opposite clear corneal incisions (OCCIs), limbal relaxing incisions (LRIs), and astigmatic keratotomy (AK) are available options for astigmatism management by modifying the corneal incision. AK is an acceptable and effective procedure for correcting astigmatism, especially in patients with pre-existing corneal astigmatism. This technique uses paired or unpaired, partial-thickness incisions of a predetermined length at the steeper corneal meridian, in order to induce flattening of the steeper meridian while steepening the flatter meridian, known as the coupling effect. Good clinical outcomes of manual AK have been reported [6, 7]. However, manual AK is often associated with unpredictable results, and its accuracy and reproducibility are limited by the depth, length, and location of the incisions. Advances in femtosecond laser-assisted cataract surgery (FLACS) technology have provided a new alternative for performing AK. Femtosecond laser astigmatic keratotomy (FSAK) is a technique that uses a femtosecond laser to make paired or unpaired, partial-thickness, arcuate incisions of a pre-specified length at the steeper corneal meridian [8, 9]. Programmed and standardized FSAK can create incisions with a precise angle, depth, and location, which can significantly improve the predictability of corneal astigmatism correction. The purpose of this study was to determine postoperative refractive and visual outcomes and astigmatic changes after FSAK in FLACS.

## Methods

### Study design and patients

This prospective interventional case series included patients at the Department of Ophthalmology, the Fourth Affiliated Hospital of China Medical University, between July 2014 and July 2015. The clinical study focused on the safety and effectiveness of FSAK; it was performed in accordance with the tenets of the Declaration of Helsinki and approved by the institutional ethics committee. Each patient was informed of the risks and benefits of the procedure and provided written informed consent. All patients underwent a detailed preoperative ophthalmologic evaluation, including slit-lamp biomicroscopy, fundus evaluation, measurement of axial length and biometry, corneal topography, and noncontact specular microscopy. Patients who presented with the following were excluded from the study: corneal astigmatism >3D, poorly dilated (< 6.0 mm) pupil, small palpebral fissure, nystagmus or obvious eyelid spasm, clear corneal leukoma, hypermature cataract, glaucoma, inflammatory or infectious pathology of the eye, or other pre-existing eye diseases, such as iris neovascularization, exfoliation syndrome, diabetic retinopathy, history of uveitis, optic atrophy, or ocular tumors.

### Surgical technique

#### Procedure for FLACS combined with FSAK

All surgeries were performed by the same experienced surgeon with the contact system LenSx (Alcon, Fort Worth, TX, USA) for laser pretreatment and Infiniti (Alcon) machine for phacoemulsification. Before surgery, all patients were treated with levofloxacin eye drops (Cravit Santen) four times daily for 3 days and pranoprofen eye drops (Pranopulin, Senju) four times daily for 1 day. On the day of surgery, patients received tropicamide and phenylephrine eye drops (Mydrin-P, Santen) for pupillary dilation (> 6.0 mm) and proparacaine hydrochloride (Alcaine, Alcon) for topical anesthesia. The patients were placed in a supine position and a speculum was placed to open the eye. Docking and suction procedures were completed by adjusting the position of the patient interface (PI) (SoftFit™) to ensure that the curved contact lens applanated the cornea. A spectral-domain optical coherence tomography (OCT) imaging device was utilized to scan the patient’s eye and locate specific target areas. After manual verification of each procedural step (corneal incisions, capsulotomy, and lens fragmentation parameters), laser treatment was performed. The patient was then transferred for the subsequent operation. After the corneal incisions and arcuate incisions were separated by blades, the anterior chamber was filled with a viscoelastic solution (Provisc; Alcon). Next, the cut anterior capsule was removed by using capsulorhexis forceps, and hydrodissection was performed. After hydrodissection, phacoemulsification of the nucleus and aspiration of the residual cortex were performed with the Infiniti phacoemulsification system. Finally, a monofocal aspheric foldable IOL (Acrysof IQ; Alcon) was implanted in the capsular bag and the corneal incisions were hydrated.

#### FSAK design

**Fig. 1:**
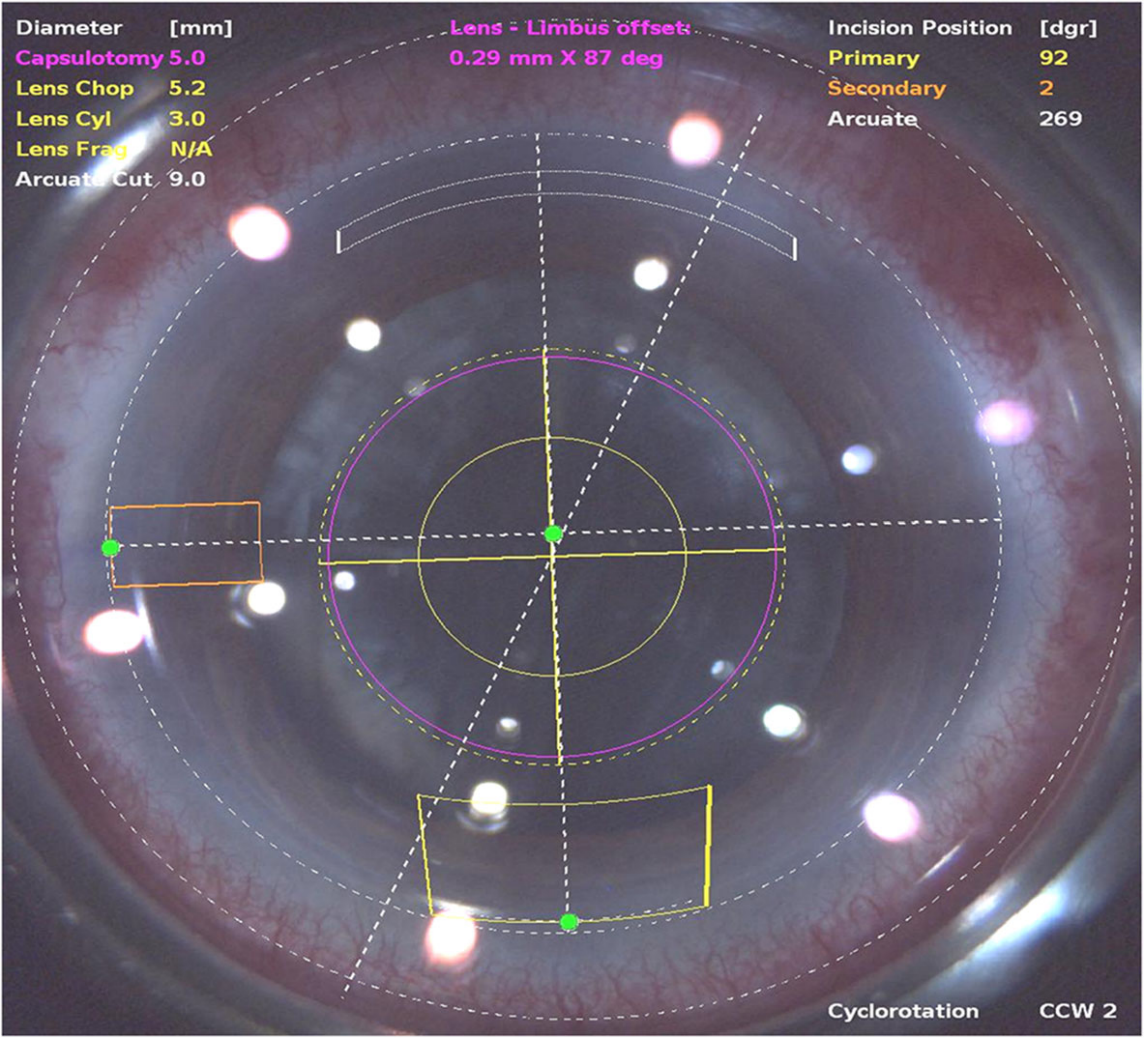
An example of the programmed femtosecond laser-assisted astigmatic keratotomy in femtosecond laser assisted cataract surgery

Combined phacoemulsification and arcuate keratotomy was performed by using the LenSx (version 2.23) femtosecond laser platform, which was guided by a real-time intraoperative spectral-domain OCT. On the basis of measurements of the corneal astigmatic axis made by preoperative corneal topography, a single arcuate keratotomy incision was paired with the 3.0-mm primary corneal incision for phacoemulsification, which was located at the corneal steep meridian. The width of FSAK was calculated online by using the Donnenfield Nomogram (http://www.lricalculator.com); a conversion arc, 9.0 mm in diameter, was needed by using LRI-incision size multiplied by 9/11. Before surgery began, the patient was seated at a slit-lamp with head aligned vertically and the corneal limbus was marked at the 0° and 180° positions with a sterile marker. The femtosecond laser energy was set at 3.0 μJ, and spot and layer separation were set at 4 μm. Keratotomy incision was placed at 85% depth of corneal thickness; the side-cut angle was set at 90 degrees at a 9.0-mm arc diameter. The primary corneal incision had a tri-planar configuration with a width of 3.0 mm, and was located at the steeper corneal meridian. One secondary incision with a width of 1.0 mm was located 90 degrees from the primary corneal incision. An example of the programmed FSAK in FLACS is shown in Fig. 1.

### Preoperative and postoperative examinations

All patients underwent examinations before surgery and 3 months after surgery, performed by the same ophthalmic technician. Preoperatively, all patients underwent an extensive ophthalmic evaluation that included slit-lamp examination, tonometry, uncorrected distance visual acuity (UDVA), corrected distance visual acuity (CDVA), manifest refraction, dilated fundoscopy, non-contact specular microscopy (SP2000P, Topcon), and corneal topography (OPD-Scan III, Nidek). At the 3-month follow up, slit-lamp examination, tonometry, UDVA, CDVA, manifest refraction, and corneal topography were repeated. Preoperative and postoperative UDVA and CDVA, manifest refraction spherical equivalent (MRSE), and the refractive and corneal astigmatisms were recorded; vector analysis of the astigmatic changes was performed by using the Alpins vector method.

### Vector analysis of astigmatic changes

Keratometric astigmatic changes were evaluated 3 months postoperatively by vector analysis with the Alpins method [10–12]. The Alpins method allows evaluation by three fundamental vectors: (1) target-induced astigmatism (TIA) vector, defined as the astigmatic change that the surgery was intended to induce, (2) surgically induced astigmatism (SIA) vector, defined as the astigmatic change that the surgery actually induced, and (3) difference vector (DV), defined as the induced astigmatic change that would enable the initial surgery to achieve its intended target.

Furthermore, relationships among these three fundamental vectors were calculated at follow-up: (1) magnitude of error (ME), defined as the arithmetic difference between the magnitudes of SIA and TIA (ME > 0 indicates overcorrection and ME < 0 indicates undercorrection), (2) angle of error (AE), which is the angle described by the vectors of SIA versus TIA (AE close to zero indicates no significant systematic error of misaligned treatment), (3) correction index (CI), the ratio of SIA to TIA (CI > 1 indicates overcorrection; CI < 1 indicates undercorrection), (4) index of success (IOS), which is the ratio of DV to TIA (indicates the success rate in correction of astigmatism), (5) flattening index (FI), the proportion of SIA that is effective in reducing astigmatism at the intended meridian.

### Statistical analysis

All descriptive statistical analyses were performed with SPSS software (version 17.0, SPSS, Inc.). The numerical data were expressed as‾*χ* ± s and the percentage data were expressed in %. A *P*-value < 0.05 was considered statistically significant. Student’s *t*-test was applied to compare refractive astigmatism, corneal astigmatism, MRSE, UDVA, and CDVA preoperatively and 3 months postoperatively. The χ^2^ test was used to analyze the rate of wearing spectacles.

## Results

This study analyzed 25 eyes of 25 patients. Of the 25 eyes, 11 (44%) belonged to men and 14 (56%) to women. The mean age of the patients was 68.56 ± 8.41 years at the time of surgery (range: 55–89 years). FLACS combined with FSAK was successfully performed on all 25 patients without incision-related complications.

### Visual acuity and astigmatic change

The mean preoperative refractive astigmatism was 1.57 ± 1.27D, which was significantly reduced to 0.70 ± 0.36D at 3 months postoperatively (*P* = 0.001). There was a statistically significant reduction in corneal astigmatism from preoperative levels (1.41 ± 0.39D) to levels (0.69 ± 0.31D) at 3 months postoperatively (*P* = 0.000). The MRSE showed no significant difference (*P* = 0.087) between preoperative and postoperative values. Statistically significant improvements in UDVA and CDVA were noted at 3 months postoperatively (*P* = 0.005 and *P* = 0.009, respectively). Table 1 shows astigmatic change and visual acuity outcomes in detail.

**Table 1:** Preoperative and 3 months postoperative astigmatic change and visual acuity outcomes

	Refractive astigmatism (D)	Corneal astigmatism (D)	MRSE (D)	UDVA (logMAR)	CDVA (logMAR)
Preoperative	1.57 ± 1.27	1.41 ± 0.39	− 1.93 ± 4.98	0.72 ± 0.23	0.40 ± 0.10
3 m-Postoperative	0.70 ± 0.36	0.69 ± 0.31	− 0.15 ± 0.91	0.13 ± 0.11	0.09 ± 0.10
*t*	3.674	10.115	−1.782	3.091	2.855
*P*	0.001^*^	0.000^*^	0.087	0.005^*^	0.009^*^

*MRSE* manifest refraction spherical equivalent, *UDVA* uncorrected distance visual acuity, *CDVA* corrected distance visual acuity

^*^P ≤ 0.05

### Rate of wearing spectacles

Three months after the surgery, only three patients (12%) needed spectacles for distant vision, which was significantly lower than the rate of 60% preoperatively (*P* = 0.001). All patients reported improvement in quality of vision and were satisfied with the treatment.

### Vector analysis of astigmatism

**Fig. 2:**
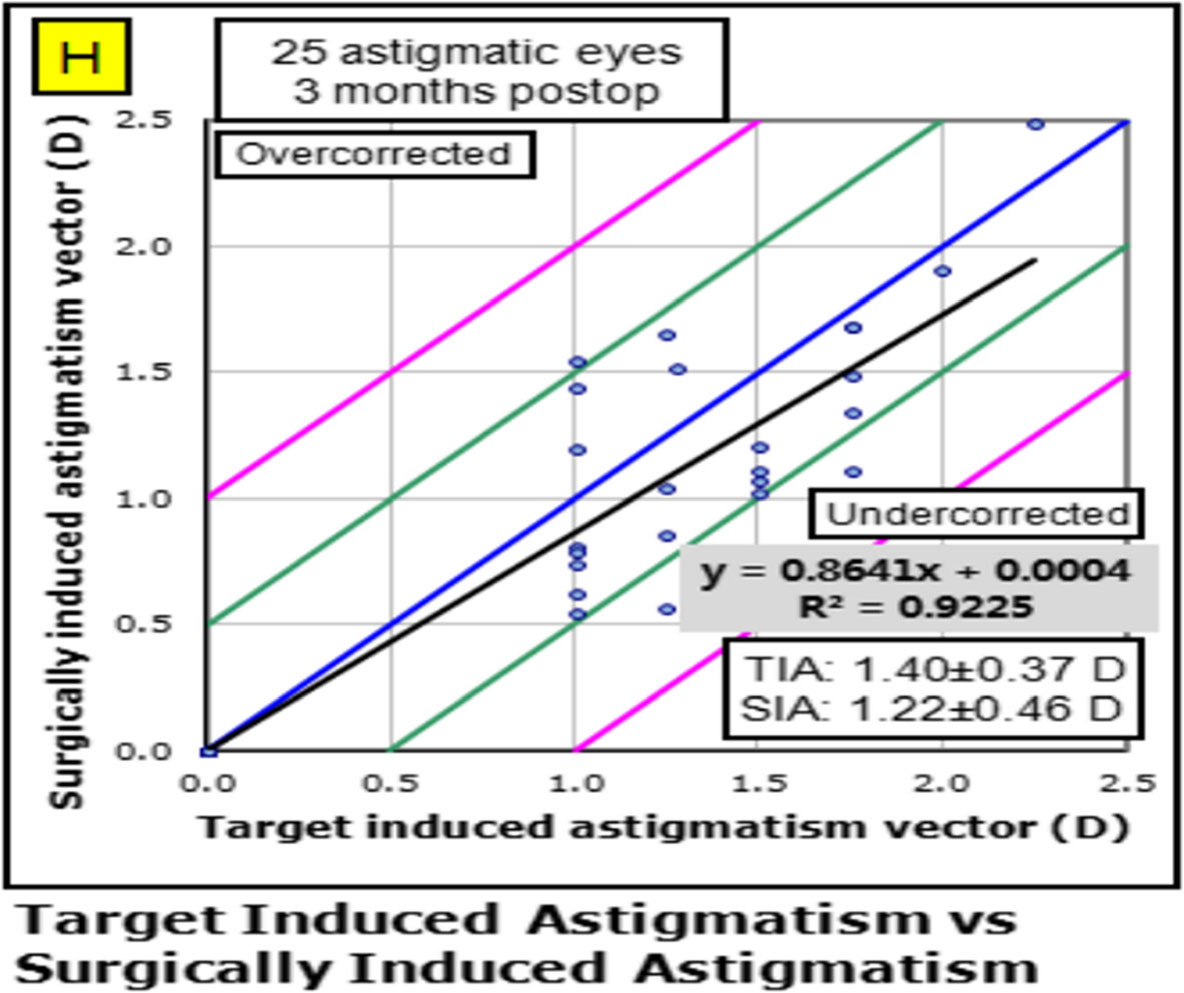
The scatter plot of target induced astigmatism vs. surgically induced astigmatism

**Fig. 3:**
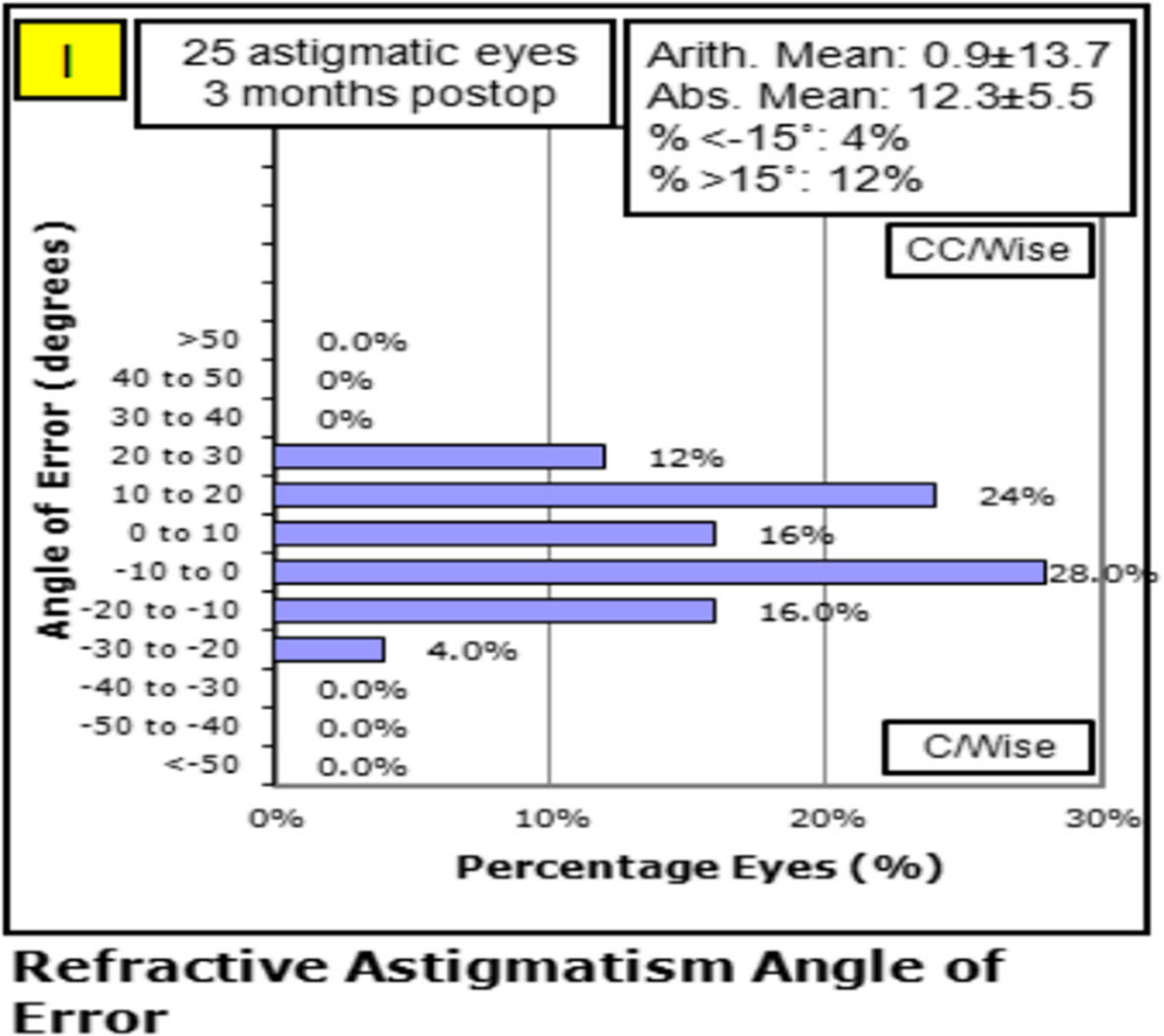
The angle of error was 0.85 ± 13.69°, indicating that the surgically induced astigmatism was counter-clockwise to the target induced astigmatism

**Fig. 4:**
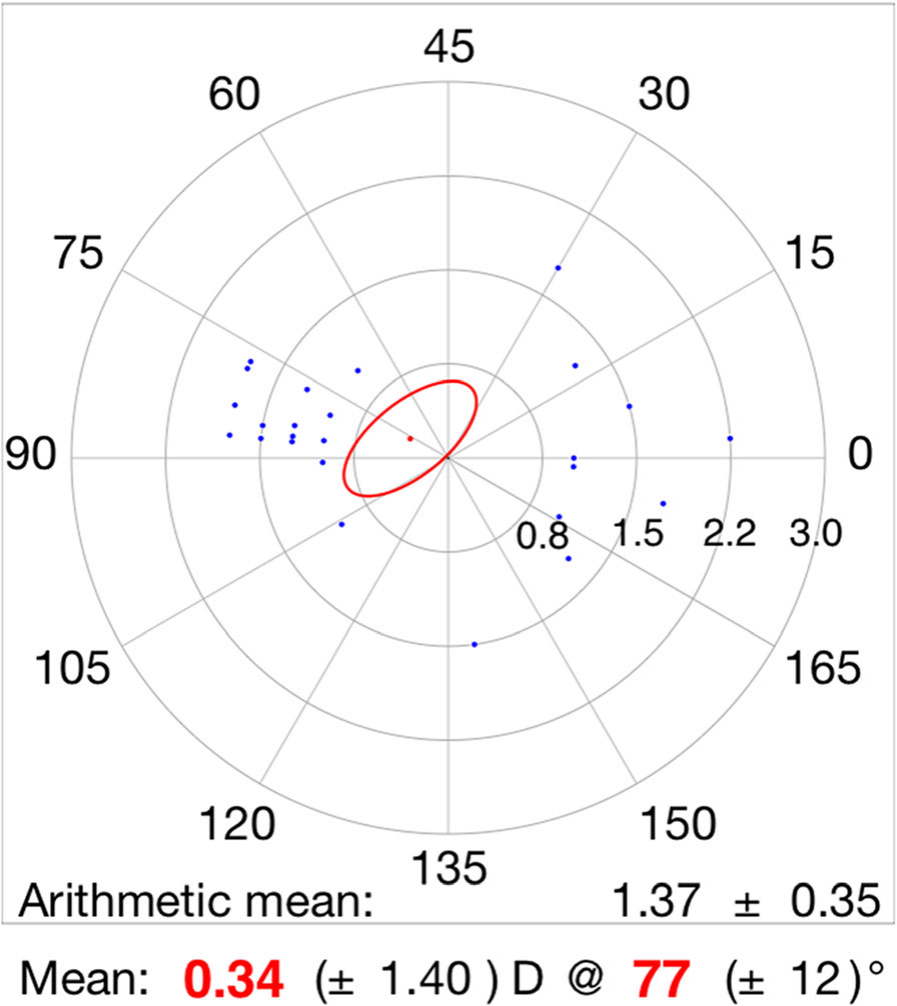
The distribution of preoperative corneal astigmatism

**Fig. 5:**
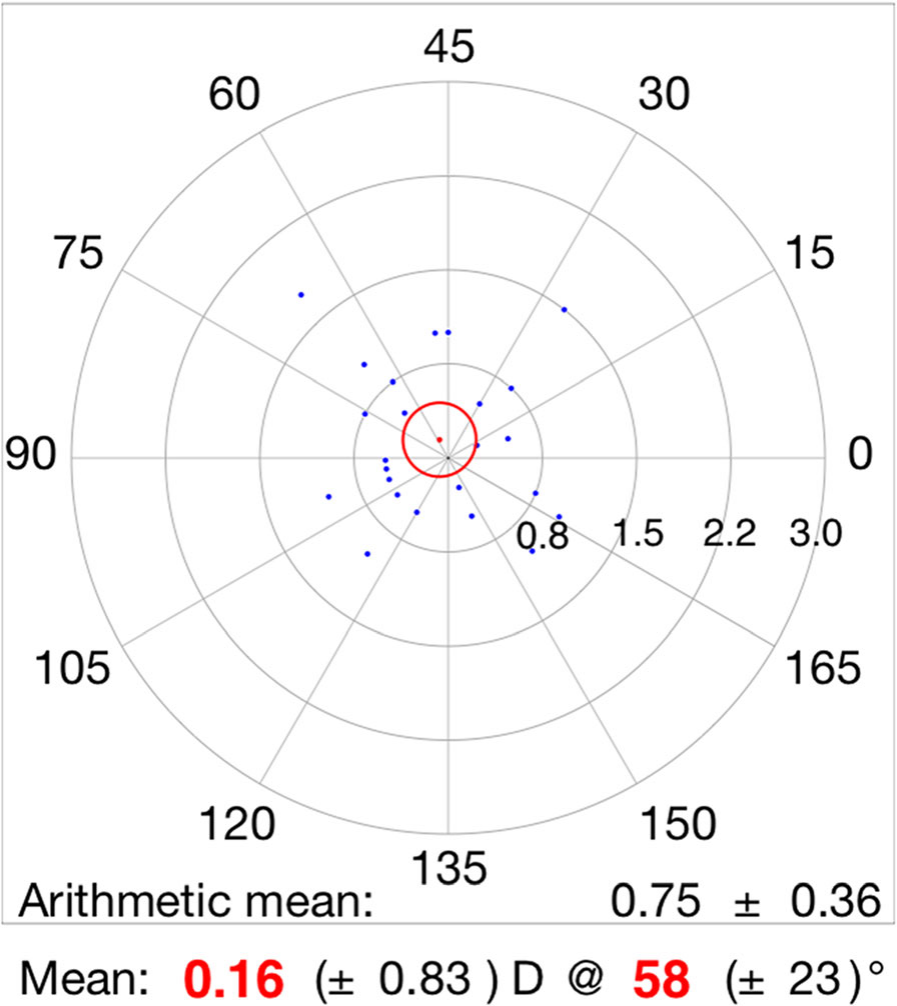
The distribution of postoperative corneal astigmatism

Table 2 shows vector analysis outcomes with the Alpins method. The mean magnitude of TIA was 1.40 ± 0.37D, whereas the mean magnitude of SIA was slightly lower: 1.22 ± 0.46D (Fig. 2). The DV was 0.70 ± 0.29D and ME was − 0.18 ± 0.36D. These numbers demonstrate a slight undercorrection 3 months postoperatively. This analysis was confirmed by the CI (0.88 ± 0.29), IOS (0.51 ± 0.19), and FI (0.78 ± 0.25) values. A scatter plot of TIA vs. SIA at 3 months after FLACS combined with FSAK is shown in Fig. 2; this plot shows undercorrection, as well as TIA that is almost >1D. The AE (Fig. 3) was 0.85 ± 13.69°, which shows that the SIA was counter-clockwise to the TIA. A wide spread of AE was noted among all eyes (horizontal axis), signifying variable alignment of flattening. The AE is positive if SIA is on an axis counterclockwise to TIA, whereas AE is negative if SIA is clockwise to TIA. Figures 2 and 3 show a combination error related to the magnitude of the treatment and the axis of the treatment. Figures 4 and 5 show the distributions of preoperative and postoperative corneal astigmatism, respectively. The postoperative centroid is closer to zero and the ellipse around the centroid is smaller than that in the preoperative figure; this represents a significant reduction in corneal astigmatism after surgery.

**Table 2:** Vector analysis outcome using Alpins method

	Mean	Range ^a^
TIA(D)	1.40 ± 0.37	1.25, 1.55
SIA(D)	1.22 ± 0.46	1.02, 1.41
DV(D)	0.70 ± 0.29	0.58, 0.82
ME(D)	−0.18 ± 0.36	−0.33, 0.04
AE(°)	0.85 ± 13.69	−4.80, 6.51
CI	0.88 ± 0.29	0.76, 0.99
FI	0.78 ± 0.25	0.67, 0.88
IOS	0.51 ± 0.19	0.43, 0.59

*TIA* target-induced astigmatism, *SIA* surgically induced astigmatism, *DV* difference vector, *ME* magnitude of error, *AE* angle of error, *CI* correction index, *FI* flattening index, *IOS* index of success

^a^95% confidence interval

## Discussion

With progress in refractive cataract surgery, demands for precision have increased among both doctors and patients. Cataract surgery can simultaneously correct refractive problems, such as myopia, astigmatism, and presbyopia, along with restoring vision and improving quality of life. However, astigmatism remains a significant obstacle in achieving emmetropia; astigmatism can produce glare, monocular diplopia, asthenopia, and visual distortions, even at relatively low levels of astigmatism. Therefore, correction of preexisting corneal astigmatism is particularly important. Astigmatism can often be managed by toric IOL implantation; several studies have evaluated the success of this technique [13, 14]. However, some patients are unwilling to choose toric IOL implantation because of the high risk of postoperative IOL rotation; alternatively, modifying corneal incisions can be performed to correct preexisting astigmatism.

There is controversy regarding the clinical understanding of various corneal incisions. AK is a technique of creating paired or unpaired partial thicknesses and incisions of a pre-specified length at the steeper corneal meridian, in order to flatten the steep meridian and reduce corneal astigmatism. The traditional manual AK incision is made closer to the corneal center, with a 6.0-mm arc diameter [15]. The traditional AK technique requires nearly flawless surgical skills and is associated with a high risk of irregular astigmatisms, which are difficult to rectify and are associated with unpredictable complications; thus, the traditional AK technique is often performed to correct only moderate-to-high astigmatisms. With the introduction of FSAK, the precision of this procedure, including arc length, depth, and location, has been greatly enhanced relative to that of manual incisions, making it suitable for low-to-moderate corneal astigmatisms. FSAK creates single or paired arcuate corneal incisions at the steeper axis of astigmatism, through femtosecond laser guidance [16, 17]. The LRIs, also known as peripheral corneal relaxing incisions (PCRIs), are made more peripherally in the cornea. These incisions are easy to create and are associated with a lower risk of irregular astigmatism and unpredictable complications, making them suitable for low-to-moderate corneal astigmatism [18]. In clinical studies of FLACS combined with corneal refractive surgery, FSAK and femtosecond laser LRI (FS-LRI) are often considered as one entity. They are both defined as arcuate corneal incisions of 8.5–9.0-mm arc diameter at the steep axis of astigmatism, made through femtosecond laser assistance. FLACS can also be combined with the femtosecond laser nonpenetrating intrastromal astigmatic keratotomy (ISAK) to correct low-to-moderate astigmatism; in this technique, the incisions are made intrastromally and retain 60–100 μm of corneal tissue anteriorly and posteriorly [19–21].

The femtosecond laser is a pulsed laser with an ultrashort pulse duration in femtoseconds (10^− 15^ s) and a near-infrared wavelength of 1030 nm. The femtosecond laser cuts tissue by vaporizing it into carbon dioxide, nitrogen, and other gases. The femtosecond laser hits its target accurately, with no damage to the surrounding tissue. Femtosecond laser-assisted surgery is also called precision medicine or precision surgery. Several clinical studies have demonstrated good refractive outcomes with different kinds of modified corneal incisions in FLACS. However, FSAK created by using a contact system (LenSx) in FLACS remains rare. Our study demonstrated that refractive astigmatism and corneal astigmatism were significantly reduced postoperatively (1.57D vs. 0.70D and 1.41D vs. 0.69D), concurrent with UDVA and CDVA improvement (logMAR values of 0.72 vs. 0.13 and 0.40 vs. 0.09). The rate of spectacle-wearing also significantly decreased 3 months postoperatively. This shows the effectiveness of FSAK in correcting preexisting corneal astigmatism in cataract surgery, consistent with other similar studies. Yoo et al. [16] showed that the refractive astigmatism decreased significantly from 1.71D to 0.78D and corneal astigmatism decreased from 1.32D to 0.87D when patients underwent FSAK of the cornea (diameter = 9.0 mm; depth = 85%) to correct post-cataract residual astigmatism by using a 60-kHz IntraLase femtosecond laser. Similar results were reported by Rückl et al. [22]; they reported that the refractive astigmatism and corneal astigmatism both decreased significantly after ISAK, from 1.41D to 0.33D and 1.50D to 0.63D, respectively. ISAK was created for paired arcuate cuts on the steep axis placed completely within the corneal stroma, with a 7.5-mm arc diameter, by an iFS femtosecond laser. At present, the clinical application of manual AK is restricted because of a lack of reproducibility of incision length and depth, a potential for axis misalignment, and wound gape associated with epithelial ingrowth into the incisions. Compared with manual AK, FSAK has many advantages to help improve the accuracy and predictability of cataract surgery. The femtosecond laser can be delivered in a specified pattern and intrastromal depth can be preoperatively set with computer software [16]. FSAK is a more accurate procedure and creates a clearer wound, leading to fewer complications; thus, it is safer than manual AK [23].

Chan et al. [24], in a retrospective case series, evaluated the outcomes of FSAK combined with cataract surgery in eyes with low-to-moderate corneal astigmatism by using the VICTUS (Bausch & Lomb, Inc.) femtosecond laser platform. FSAK showed greater reduction of refractive and corneal astigmatism, 1.33D vs. 0.87D and 1.23D vs. 0.81D; vector analysis demonstrated slight undercorrection. Our study showed that the DV was 0.70 ± 0.29D and ME was − 0.18 ± 0.36D, with a CI of 0.88 ± 0.29. Undercorrection was also detected in our study. The meridian of primary corneal incisions may differ because of patient-specific differences in the steep axis, which may result in a difference between TIA and SIA. Compared with temporal corneal incisions, inferior corneal incisions can cause greater SIA, approximately 0.5D [25]. An unpredictable increase in FSAK incision depth during femtosecond laser pretreatment may also result in a difference between TIA and SIA. Further, it may be related to the offset laser caused by corneal folds or unexpected eye movement. In a study by Nejima et al. [26], anterior segment OCT showed that the depth of FSAK had deepened by 60 μm more than the specified setting. Mayer et al. [27] compared inflammatory cell response and morphological aspects of femtosecond laser-created corneal incisions; they found no differences in corneal inflammatory cell response, but observed a saw-tooth-like cutting edge and a significantly higher cell death rate than in the case of manually performed incisions, indicating an upregulated postoperative wound-healing response [28]. These may affect the healing process and biomechanical action, thereby causing an unpredictable correcting effect. Moreover, the incision location may change slightly due to eyeball rotation. In our study, the patient was seated at a slit-lamp and the corneal limbus was marked to improve accuracy. However, some errors remain in manual limbus marking, which can be resolved by the introduction of a cataract surgery navigation system. This system is the integration of multiple modules and functions, including preoperative measurement, surgery design, intraoperative navigation, and combination with femtosecond laser. In our previous studies, we found that corneal incisions without separation were self-sealing and had little effect on corneal curvature changes. Hence, we suggest that arcuate incisions ought to be separated by blades to achieve better refractive outcomes. However, sufficient quantitative indicators were not available to determine the necessity of FSAK manual separation in the present study: thus, this merits further research.

Our study demonstrated that corneal astigmatism decreased, from 1.41D preoperatively to 0.69D postoperatively. Compared with the study by Chan et al. [24], the CI in our study was slightly higher (0.88 ± 0.29 vs. 0.86 ± 0.52) and the IOS in our study was much closer to zero (0.51 ± 0.19 vs. 0.62 ± 0.45). Thus, our study demonstrated more predictable outcomes. The variability of the inconsistent outcomes of these two different pretreatment procedures may be the result of discrepancies in arc diameter and depth of FSAK incisions. We set parameters of a 9.0-mm arc diameter and 85% depth of corneal thickness, whereas Chan et al. set parameters of 8.5-mm arc diameter and 450-μm depth. Notably, FSAK incisions are deeper and more peripheral. We used a standard arcuate incision combined with only one primary and secondary incision each, rather than two 1.5-mm paired secondary incisions, which could also contribute to the variability in treatment effects. We set 3.0 μJ as the femtosecond laser energy, which was higher than the 1.7 μJ energy used by Chan et al.; this may have led to a stronger laser delivery effect. Additionally, analysis of results at 3 months postoperatively in our study may reveal more stable outcomes than at 2-months postoperatively. Finally, the arcuate keratotomy was separated with blades in our technique, although the duration of wound healing and stabilization remains to be elucidated in further studies.

## Conclusions

In conclusion, the use of FSAK in FLACS was effective and promising for eyes with pre-existing corneal astigmatism. However, the combination of FLACS and FSAK can achieve more accurate and consistent outcomes through accurate preoperative topographic measurement and surgical design, sufficient education of patients to improve cooperation during laser pretreatment, adequate surgical experience and skill, and the use of cataract surgery navigation systems, such as the Zeiss Callisto Eye (Carl Zeiss AG, Dublin, CA, USA) and the Alcon Verion Image Guided System (Alcon) [29]. The limitations of the current study are its small sample size and 3-month short-term follow-up. Further work is required to verify the long-term refractive outcomes and refractive stability in large populations of cataract surgery candidates.

## Abbreviations

AE: Angle of error
AK: Astigmatic keratotomy
CDVA: Corrected distance visual acuity
CI: Correction index
D: Diopters
DV: Difference vector
FI: Flattening index
FLACS: Femtosecond laser-assisted cataract surgery
FSAK: Femtosecond laser astigmatic keratotomy
IOL: Intraocular lens
IOS: Index of success
ISAK: Intrastromal astigmatic keratotomy
LRIs: Limbal relaxing incisions
ME: Magnitude of error
MRSE: Manifest refraction spherical equivalent
OCCIs: Opposite clear corneal incisions
OCT: Optical coherence tomography
PCRIs: Peripheral corneal relaxing incisions
PI: Patient interface
PIs: Primary corneal incisions
SIA: Surgically induced astigmatism
TIA: Target-induced astigmatism
UDVA: Uncorrected distance visual acuity
